# Knowledge Gaps and Determination of Attitude and Practice among Medical Students toward Hepatitis B Infection: A Nationwide Cross-Sectional Study

**DOI:** 10.1155/2024/2730516

**Published:** 2024-03-21

**Authors:** Nader Alaridah, Raba'a F. Jarrar, Rayan M. Joudeh, Haneen Al-Abdallat, Layan Ismail, Zaina Alnajjar, Mohammad Jum'ah, Anas Haidar Abu-Humaidan

**Affiliations:** ^1^Department of Pathology, Microbiology, and Forensic Medicine, School of Medicine, University of Jordan, Amman 11942, Jordan; ^2^Department of Clinical Laboratory Sciences, School of Science, University of Jordan, Amman, Jordan; ^3^College of Medicine, Sulaiman Al-Rajhi University, Al-Bukayriah, Al-Qassim, Saudi Arabia; ^4^School of Medicine, University of Jordan, Amman 11942, Jordan; ^5^Faculty of Medicine, Hashemite University, Az Zarqa, Jordan; ^6^Faculty of Medicine, Al-Balqa Applied University, As-Salt, Jordan

## Abstract

**Introduction:**

As reported by the World Health Organization (WHO), annually, there are 1.5 million new infections, with more than 290 million people living with chronic hepatitis B diseases in 2019. Medical students (MSs), a subgroup of health care workers (HCWs), are at high occupational risk for HBV infection since HCWs have a 2–10 times greater risk of getting the virus than the general population. Therefore, they must have a broad understanding of hepatitis B infection. In this study, we aimed to assess the level of knowledge, attitude, and practices among MSs, and to explore its predictors. *Methodology*. A descriptive cross-sectional research was done among MSs, those who finished their infectious disease course. MSs were asked to participate in a self-administered online-structured questionnaire.

**Results:**

612 MSs were enrolled in our study. 67.5% were females, and 36.9% were in their 6th year. Around half of the participants held a high level of knowledge, attitude, and practices toward HBV infection. MSs in the last year of the study who encountered HBV patients showed to have better knowledge. Male MSs who encountered HBV patients and took extra HBV courses showed better attitudes. High practice level among MSs was associated with being in the 6th year and taking extra HBV courses.

**Conclusions:**

This study demonstrated a satisfactory level of knowledge, attitude, and practices toward HBV infection among MSs. However, awareness must be enhanced in some areas especially transmission routes of HBV. Thus, it will strengthen the level of attitude and practices to omit the effect of the stigma while delivering care to HBV patients.

## 1. Introduction

Hepatitis B virus (HBV) infection is a critical global health issue that causes both acute and chronic hepatitis with significant morbidities including liver cirrhosis and hepatitis-induced liver cancer [1]. As reported by the World Health Organization (WHO), annually, there are 1.5 million new infections, with more than 290 million people living with chronic hepatitis B diseases in 2019 [2]. HBV is mostly transmitted by contact with an infected person's blood and other bodily fluids [2, 3], through sharing needles during injections, reusing contaminated needles, unprotected sexual contact, and perinatal transmission (transfer from an infected mother to a baby) [4–7]. Medical students (MSs), a subgroup of health care workers (HCWs), are at high occupational risk for HBV infection since they have a 2–10 times greater risk to get the infection compared to the general population [8–10]. Lack of competence and professional ability increases the risk of HBV infection [11]. Furthermore, screening and vaccination are effective preventative approaches for individuals who are at risk of HBV infection [2]. In 2017, the prevalence of HBV infection in Jordan reached 230,000 cases, and only about 0.7-0.9% of those received treatment. This indicates a lack of appropriate health and follow-up for infected individuals [12]. Since the beginning of the pediatric vaccination program in 1995, the prevalence of HBV infection has declined from 9.9% in 1985 to 2.4% in 2016 [13–15]. In 2017, according to the Jordanian HBV Working Group Meeting conducted in the United Arab Emirates, the general awareness level toward HBV among the Jordanian population was ranked low to medium, as well as it showed low levels for related factors such as illness prevalence, origin, transmission, and high-risk categories [12].

Clearly, a lack of awareness among HCWs regarding HBV has been observed, potentially raising the risk to contracting the disease [16]. Thus, MSs' understanding of HBV is crucial, and undergraduate HBV education should be addressed since students may have substantial exposures and are thus most at risk of infection [17]. Furthermore, MSs are potential future physicians and leaders, and these students need to understand the epidemiology, determinants, screening, and management of HBV to promote effective prevention, early diagnosis, and successful treatment [11]. However, the level of awareness toward HBV infection among undergraduate MSs varies globally [18–21]. Consequently, it is important for the medical colleges to identify areas of weakness in MSs and actively contribute to the training and development of the future generations of medical practitioners. Given the important role that MSs play in the healthcare system, a thorough understanding of HBV infection is vital. To date, there is a lack of studies exploring the knowledge, attitude, and practices (KAP) and the associated factors among MSs in Jordan.

## 2. Methodology

A cross-sectional study was conducted among MSs and reached responses from 612 participants during the time period of March and August 2022. Our target population consisted of MSs who completed the infectious diseases courses in the third year (i.e., 3rd year to 6th year) from the following universities: University of Jordan, Mut'ah University, Jordan University of Science & Technology, Hashemite University, Al-Balqa Applied University, and Yarmouk University. Participants from schools other than medical school and the first-second year MSs who have not finished infectious diseases course were excluded. Participant-driven sampling method was used to recruit students from all medical schools. The questionnaire was distributed using Google Forms to eligible individuals through student representatives at each university via messages and placed in the main official social media groups for each group of participants. These groups are private and supervised by a student representative from the relevant university. The study requires a minimum sample size of 385 people, which was estimated using a 5% margin of error and a 50% prevalence. [22] This study is part of a wider national study conducted in Jordan and aimed at assessing the level of knowledge, attitudes, and practices toward HBV infection among healthcare students in Jordan [23].

### 2.1. Questionnaire Administration

Eligible MSs were invited to participate voluntarily to fill out the questionnaire. The questionnaire was sent to the participants through an online survey link. All subjects were consented to enroll in the study. It was explicitly mentioned that participants could withdraw their responses at any time without penalty. Participants were requested to submit the necessary information to obtain reliable results, but no personal information was gathered.

### 2.2. Measurement Tool

Based on previously validated surveys, a structured English self-administered online questionnaire was used [24, 25]. The questionnaire was divided into four sections: participant demographics with 7 questions, knowledge section with 43 questions, attitude section with 8 questions, and practices section with 3 questions. A pilot test was done on the questionnaire to make sure the questions were clear and comprehensible before they were distributed and used. The questionnaire is included in supplementary file 1.

### 2.3. Ethical Considerations

The protocol for the study was developed in accordance with the Helsinki Declaration's ethical standards, and it was reviewed and approved by the Institutional Review Board (IRB) at the University of Jordan, on 1/25/2022 (reference number: 1/2022/2506) in meeting no. 2022/1. All individuals gave their consent before completing the questionnaire. The information was obtained and analyzed in confidence before being saved on a personal computer that only the authors have access to.

### 2.4. Statistical Analysis

Microsoft Excel 2016 was used to enter the data, which was then imported into IBM SPSS version 25 (IBM Corp., Armonk, New York, USA) for analysis. Descriptive statistics were analyzed and reported as either frequency and percentage or mean and standard deviation for each numerical and categorical variable, respectively. The Chi-square test was used to assess the relationship between demographic factors, knowledge, attitude, and practices. Multivariate regression analysis was used to evaluate each independent variable after controlling for possible confounders. Each correct response received one point. If a participant correctly responded to 70% of the questions or more in each KAP section, the score was deemed to be good. If less than 70% of the questions in each section were correctly answered, the participant's score was deemed poor.

## 3. Results

### 3.1. Demographics of Survey Participants

As illustrated in Table 1, 612 participants were enrolled. The mean age was 22.4 years, and 67.5% of them were females. Most participants (36.9%) were in the sixth year of study, followed by those in the third year (32.7%), fourth year (21.1%), and fifth year (9.3%). The majority of MSs (91.2%) have not taken additional HBV courses besides the university lectures. During the clinical training, about 32.8% of participants encountered hepatitis B patients.

### 3.2. Knowledge toward the Hepatitis B Virus

15% of MSs were aware of the prevalence of HBV infection in Jordan. Also, three-quarters (76.6%) of them were aware of HBV infection sequalae. Only 11.4% agreed with the serious dangers of dying prematurely in the absence of appropriate treatment and follow-up. Furthermore, 17.5% understood that newborns had the greatest risk of developing chronic HBV infection after initial infection, whereas only 16% were aware that vertical transmission at birth is the main reason for HBV infection in Jordan. As shown in Table 2, MSs in the survey showed good knowledge regarding HBV transmission routes. Most of them correctly answered as follows: HBV may be transmitted by blood donation (97.7%), vertically at birth (88.2%), or via unprotected sexual intercourse (83.7%). Whereas MSs correctly answered that HBV cannot be spread by sneezing or coughing (78.4%), and 86.8% properly acknowledged that HBV cannot be transmitted through a handshake. However, 44.1% still incorrectly believed that HBV might be transferred through the exchange of utensils used by HBV-infected patients. For HBV infection prevention strategies, MSs were aware of the following: 89.2% for hepatitis B vaccination, 82.2% for condom usage, and 96.7% for avoiding the reuse or sharing of needles and syringes. Whereas 49.8% and 47.1% of MSs mentioned that meticulous cleaning and meal preparation and avoiding sharing food and equipment are ineffective in preventing the spread of infection, respectively. 50% of MSs agreed on the need for sharp-proof containers for disposing of needles in clinics and hospitals. Approximately 23.5% of MSs were aware that immunizing neonates at birth might reduce perinatal transmission, and 13.9% of MSs were aware that the first dose of hepatitis B vaccination should be administered within 24 hours of delivery. 42% of MSs knew that the most efficient method of preventing children from getting HBsAg from infected mothers is to provide the first dose of the hepatitis B vaccination and hepatitis B immune globulin (HBIG) within 48 hours of delivery and to finish the vaccine series. Half of the MSs (50.5%) understood that the hepatitis B surface antigen (HBsAg) test is used to confirm HBV infection, and only 51.5% knew that the hepatitis B surface antibody (anti-HBs) test is used to test immunity toward HBV. Only 3.8% of MSs were aware that 12 months is the appropriate age for screening for HBV infection in newborns whose mothers have positive HBsAg. MSs' awareness of HBV infection symptoms was limited, with only 9.2% of MSs correctly identified that most patients are without symptoms. 45.4% of MSs were aware about the measures to identify the necessity and the need for therapy in patients with HBsAg. Although 56.9% of those polled were aware that hepatitis B is incurable, there are effective drugs that can aid with illness management and control. Table 2 shows that most participants are familiar with the objectives of HBV therapy. Around 55.4% of MSs were unaware that nucleotide analogs (NAs) are the first-line and long-term therapy for HBV infection. Only 29.2% were aware that not all patients with HBV infection require prompt treatment and that only those with severe liver damage or cirrhosis do. However, regardless of treatment necessity, monitoring is a critical step for all HBV patients, and 67.2% of MSs were aware of this.

### 3.3. Attitudes toward the Hepatitis B Virus

As illustrated in Table 3, approximately 51.3% of survey MSs were comfortable advising patients on HBV prevention. Furthermore, 67.3% and 66.8% felt comfortable with conducting lab diagnostic and monitoring tests, respectively, while just 34% of survey MSs felt confident prescribing drugs to hepatitis B patients. 84.6% of MSs agreed with the safety of HBV vaccination, and 62.3% believed that neonates should be immunized against HBV at birth. Concerns were stated by about 38.7% regarding contact transmission or being in the same area, and 31.4% regarding eating food with and/or sharing a meal with hepatitis B patients.

### 3.4. Practice toward Hepatitis B Virus

Before starting a clinical internship in a healthcare system, 68.3% received hepatitis B vaccines, and 42.6% underwent hepatitis B testing. 42.3% of MSs said that they always use gloves when giving injections or doing medical operations. These results are illustrated in Table 4.

### 3.5. Associated Factors toward Knowledge, Attitude, and Practices toward HBV Infection

The association between sociodemographics and the level of KAP is shown in Table 5.

In multivariate analysis, some variables were associated with high-level knowledge among MSs, including studying year level, 6th-year students (OR: 7.589, Cl: 3.469-16.601, *p* ≤ 0.001, ref: 3rd year), and any previous encounter of hepatitis B patients (OR: 1.667, Cl: 1.114-2.494, *p* value: 0.013, ref: no). In addition, the following variables are associated with a positive attitude: males (OR: 1.557, Cl: 1.088-2.229, *p* value: 0.016, ref: females), 6th-year students (OR: 1.862, Cl: 0.891-3.891, *p* value: 0.098, ref: 3rd year), encountering hepatitis B patients (OR: 2.249, Cl: 1.517-3.334, *p* ≤ 0.001, ref: no), and any extracurricular courses taken (OR: 2.234, Cl: 1.174–4.250, *p* value: 0.014, ref: no). Variables associated with better practices are as follows: 6th-year students (OR: 2.479, Cl: 1.178-5.218, *p* value: 0.017, ref: 3rd year), any previous encounter with hepatitis B patients (OR: 1.262, Cl: 0.849-1.878, *p* value: 0.250, ref: no), and any extra courses completed about HBV (OR: 4.104, Cl: 1.983–8.494, *p* ≤ 0.001, ref: no). Multivariate analysis results are illustrated in Table 6.

## 4. Discussion

The WHO has set a goal of viral hepatitis elimination by 2030 [26], but the gap in general knowledge and awareness seems to be a barrier to reach this important goal [27]. Our study ought to evaluate KAP toward HBV infection among MSs in the clinical years in Jordan since there is no available data regarding it. Results of the study showed an overall satisfactory level of KAP toward HBV infection among the 612 MSs. In addition, predictors associated with a high level of KAP were noticed. The results of this study showed a high level of knowledge of hepatitis B infection among MSs (54.5%), which is consistent with the results reported among MSs in studies in Ethiopia, Ghana, Senegal, Saudi Arabia, and Nepal [19–21, 28], and these findings demonstrate the MS's solid foundation in HBV-related knowledge, which is critical for future healthcare professionals who will be at the forefront of patient treatment. However, the result was inconsistent with studies done in Syria and Ethiopia [11, 18]. In this study, MSs had poor knowledge regarding prevalence of HBV infection, the high-risk group, and the consequences of neglected HBV infection (<17.5%). These results are in contrast with a study conducted in Vietnam and Saudi Arabia [25, 28], where the MSs were aware of the consequences of the HBV infection on the health. In terms of transmission routes, the knowledge was high which is consistent with other studies [11, 18, 19, 21, 28]. However, surprisingly, we have noticed that 44.1% of MSs claimed that HBV infection can be transmitted by sharing food and utensils. This is an alarming finding, which can have an impact on the level of stigma with encountering people who are infected by HBV, which has been reported by a study in Saudi Arabia among MSs [28], and a similar study in Iraq showed that MSs had a negative attitude regarding sitting and shaking hands with people who were infected by HBV [29], which is in contrast to Nepal study among undergraduate MSs [30]. In addition, a previous study in Jordan found a significant level of stigma among nurses while delivering care to a patient infected by HBV [31]. These gaps underline the importance of focused educational initiatives to address specific features of HBV transmission, and targeted training programs might improve students' understanding of these essential topics. The overall knowledge regarding the prevention of HBV infection was moderate. MSs have identified the general preventive measures such as the importance of vaccination and identifying the high-risk group. In addition, they were aware of the HBV transmission preventive measures such as avoiding using needles and using condoms as reported in another study [32]. However, few gaps were noticed in vaccination as preventive measures. 13.9% were aware of the time for the first dose of the hepatitis B vaccine for babies, and less than one-quarter of the MSs (23.5%) were aware of the prevention of HBV perinatal transmission. Knowledge regarding the treatment of HBV infection was moderate, and knowledge about antiviral regimens and their courses should be improved among MSs in Jordan. Attitude toward treating HBV infection and dealing with people who have hepatitis B was assessed. Our study reported that 51.3% of MSs had a high level of attitude toward HBV infection, similar to a study conducted in Senegal [21]. It is worth to mention that around two-thirds of MSs (66%) were not confident in treating a patient with HBV infection, and this is consistent with a study in Senegal [21]. MSs did not identify the antiviral medications used for HBV treatment. In addition, regarding MS' attitude toward discrimination and stigma with HBV infection, two-thirds of MSs were concerned about contacting HBV patients during the casual event (61.3%) as well as sharing food and utensils (68.6%) with them; studies in Vietnam and Saudi Arabia reported the same result [25, 28]. Strong et al. affirmed that “misunderstanding of the transmission route may strengthen the stigma against individuals with HBV” [33]. Therefore, improving transmission route knowledge would result in more understanding of the pathways of getting HBV infection. Eventually, controlling the level of stigma in MSs would impact the level of care provided to HBV-infected patients. Overall practices' level toward HBV infection among MSs is high (53.1%). More than two-thirds of the students (68.3%) received the HBV vaccine before entering clinical practice similar to results of other studies conducted in Senegal and Saudi Arabia [21, 28], but Jordanian MS's vaccine administration is less than their fellow students in Nepal and Iran [30, 32], while around forty percent of MSs were screened for HBV before clinical practice [11]. Only 42.3% of MSs consistently use gloves before contacting patients or performing medical procedures, which is lower than a study conducted in Vietnam [25]. In this study, the female gender was related to a positive attitude. High levels of education were linked to high levels of knowledge and practice. Encountering patients with HBV infection was confirmed to be a predictor of high levels of knowledge and attitude. Extra HBV courses were shown to be related to high levels of attitude and practice. Furthermore, MSs are at a high risk of needle stick injury and are exposed to blood in their professional practice, and these are critical concerns that must be addressed via education. Furthermore, it is advised that a protocol should be established for appropriate vaccination and infection prevention instruction for all MSs before they begin clinical years.

### 4.1. Strengths and Limitations

To the best of our knowledge, this study surveyed a representative sample of MSs from all prospective medical schools in Jordan's universities, ensuring considerable representation and facilitating data extraction. Our study has few limitations such as the 5th-year respondents were very less in comparison with respondents from other years and the participant-driven sampling method that we used could have attributed to this disproportionate number of participants in one arm, as well as the 5th-year medical students have busy and condensed schedule most of the year making it difficult to reach them. There are few self-reported data by participants in this study that cannot be verified, such as HBV testing and vaccination. Furthermore, establishing a cause-and-effect relationship from a cross-sectional study is difficult.

## 5. Conclusion

According to the findings of this study, more than half of the MSs had an unsatisfactory level of knowledge, attitude, and practices about hepatitis B infection. The female gender was related to a positive attitude. High levels of education were linked to high levels of knowledge and practice. Encountering patients with HBV infection was discovered to be a predictor of high levels of knowledge and attitude. Extra HBV courses were shown to be connected with high levels of attitude and practice. Awareness must be increased, particularly for HBV mode of transmission, diagnostic procedures, and treatment approaches. Thus, strengthening the levels of attitude and practice omits the impact of stigma while delivering care to HBV patients.

## Figures and Tables

**Table 1:** Demographics of survey participants.

Respondents' demographics		*n*	%
Age mean	22.4		

Gender	Male	199	32.5%
	Female	413	67.5%

University	JU	109	17.8%
	Hashemite	102	16.7%
	Yarmouk	100	16.3%
	JUST	99	16.2%
	BAU	100	16.3%
	Mut'ah	102	16.7%

Studying year	3rd year	200	32.7%
	4th year	129	21.1%
	5th year	57	9.3%
	6th year	226	36.9%

Have you taken any extra HBV courses (besides your university lectures)?	Yes	54	8.8%
	No	558	91.2%

Have you encountered any CHB patient before?	Yes	201	32.8%
	No	411	67.2%

**Table 2:** Knowledge about HBV.

Questions	Correct answer	
Prevalence and sequelae		
A4. What percentage of the Jordanian population has chronic hepatitis B (CHB)?	92	15.0%
A5. How did most people who have CHB in Jordan get infected?	98	16.0%
A6. Which age group is most likely to develop CHB after the initial infection?	107	17.5%
A7. What are the consequences of chronic hepatitis B?	469	76.6%
A52. Without proper monitoring and treatment, what is the chance a patient would die of CHB complications?	70	11.4%
Transmission routes		
A8. Can hepatitis B be transmitted through handshake?	531	86.8%
A9. Can hepatitis B be transmitted through unprotected sex?	512	83.7%
A10. Can hepatitis B be transmitted through blood transfusion?	598	97.7%
A11. Can hepatitis B be transmitted through sneezing or coughing?	480	78.4%
A12. Can hepatitis B be transmitted from mother to child at birth?	540	88.2%
A13. Can hepatitis B be transmitted through sharing food or utensils?	342	55.9%
Prevention		
Prevention measures		
A14. Can cleaning and cooking food thoroughly prevent HBV transmission?	305	49.8%
A15. Can the hepatitis B vaccine prevent HBV transmission?	546	89.2%
A16. Can HBV transmission be prevented by not reusing or sharing needles/syringes?	592	96.7%
A17. Can HBV transmission be prevented by avoiding sharing food/utensils or eating with a person with chronic HBV?	288	47.1%
A18. Can using a condom prevent HBV transmission?	503	82.2%
A19.What is the most effective preventive measure for infants born to mothers with chronic HBsAg?	257	42.0%
A21. Who needs the hepatitis B vaccine?	495	80.9%
A23. Prevention of mother-to-child transmission	144	23.5%
A24. The first dose of hepatitis B vaccine for baby	85	13.9%
A33. Is it necessary to have sharp-proof containers at clinics for disposing of needles and sharp objects? What would you do to prevent needle stick injury?	306	50.0%
Diagnosis and treatment		
Who should be tested for hepatitis B?		
A35. Pregnant women should be tested for hepatitis B	429	70.1%
A36. HIV-infected people should be tested for hepatitis B	524	85.6%
A37. Men who have sex with men (MSM) should be tested for hepatitis B	429	70.1%
A38. Family members of those who have hepatitis B should be tested for hepatitis B	515	84.2%
A56. Serum HBsAg test for identification of patients infected with hepatitis B virus	309	50.5%
A57. What test should be used to identify immunity against the hepatitis B virus?	315	51.5%
Diagnosis		
A40. What is the symptom most patients with chronic hepatitis B present?	56	9.2%
A41. What are the criteria for indicating treatment in patients with CHB?	278	45.4%
A55. When should infants born to mothers with CHB be evaluated for HBsAg status?	23	3.8%
Treatment		
A42. There is no cure, but there are effective medications to manage and control the disease	348	56.9%
Treatment goal: What are the treatment goals for CHB patients?		
A43. Inhibit the replication of the hepatitis B virus	530	86.6%
A44. Prevent disease progression of disease, particularly liver cirrhosis and liver cancer	566	92.5%
A45. Prevent mother-to-child transmission (MTCT)	537	87.7%
A46. Prevent flare of hepatitis B	526	85.9%
Treatment principles		
A47. Is it true that nucleos(t)ide analogs (NAs) are a recommended first-line treatment for CHB?	273	44.6%
A48. Is treatment of CHB with NAs long term, possibly even lifetime?	266	43.5%
A49. Do patients need to strictly adhere to the treatment of CHB?	478	78.1%
A50. Do you think that all patients with chronic HBV need to be treated immediately?	179	29.2%
A51. Should all CHB patients be monitored and tested regardless of treatment status?	411	67.2%

Abbreviation: CHB: chronic hepatitis B; HBV: hepatitis B virus; MSM: men who have sex with men; MTCT: mother-to-child transmission; NAs: nucleos(t)ide analogs.

**Table 3:** Attitude about HBV.

Questions	Correct answer	
	*n*	%
A20. Are you confident in counseling patients about prevention of HBV?	314	51.3%
A22. Do you think that the hepatitis B vaccine is safe?	518	84.6%
A25. Do you think it is necessary to vaccinate newborns for hepatitis B at birth?	381	62.3%
A53. Are you confident in ordering laboratory tests to monitor CHB patients?	409	66.8%
A54. Are you confident in prescribing treatment for a patient with chronic hepatitis B?	208	34.0%
A58. Are you confident in ordering diagnosis tests for patients with chronic HBV?	412	67.3%
A59. Would you have any concerns having casual contact or working together with a chronic HBV patient in the same office?	237	38.7%
A60. Would you have any concerns sharing food or utensils with a CHB?	192	31.4%

**Table 4:** HBV preventive practices.

Questions	Correct answer	
	*n*	%
A28. Did you get the hepatitis B vaccine before entering practicum at teaching hospitals?	418	68.3%
A29. Did you get tested for HBV before entering practicum at teaching hospitals?	261	42.6%
A34. Do you consistently wear gloves when administrating injections or performing medical procedures to patients?	259	42.3%

**Table 5:** Knowledge, attitude, and practices toward HBV and its associated factors.

		Level of knowledge			Level of attitude			Level of practice		
		High K	Low K	*p* value	High A	Low A	*p* value	High P	Low P	*p* value
Gender	Male	107	92	0.781	115	84	.026	103	96	.643
	Female	227	186		199	214		222	191	

Studying year	3rd year	71	129	. ≤0.001	80	120	≤0.001	83	117	≤0.001
	4th year	57	72		62	67		53	76	
	5th year	39	18		28	29		34	23	
	6th year	167	59		144	82		155	71	

History of HBV infection	Yes	4	2	0.550	5	1	0.115	4	2	0.504
	No	330	276		309	297		321	285	

Family history of HBV infection	Yes	14	6	0.159	14	6	0.089	10	10	0.777
	No	320	272		300	292		315	277	

Extra courses about HBV	Yes	31	23	0.662	39	15	≤0.001	44	10	≤0.001
	No	303	255		275	283		281	277	

Encountered HBV patient	Yes	141	60	≤0.001	137	64	≤0.001	129	72	≤0.001
	No	193	218		177	234		196	215	

*p* value by univariate logistic regression.

**Table 6:** Logistic regression analysis between knowledge, attitude, and practice toward HBV infection among MSs.

Covariates	Knowledge			Attitude			Practice		
	OR	CI	P-value	OR	CI	*p*-value	OR	CI	*p* value
Age	0.855	(0.726-1.008)	0.062	1.001	(0.855-1.172)	0.991	1.032	(0.880-1.211)	0.697
Gender	NA			1.557	(1.088-2.229)	0.016	NA		
Male									
Female				Reference					
Studying year									
3rd year	Reference			Reference			Reference		
4th year	1.511	(0.912-2.504)	0.109	1.131	(0.684-1.870)	0.631	0.919	(0.554-1.524)	0.743
5th year	4.433	(2.169-9.062)	≤0.001	1.090	(0.550-2.158)	0.805	1.858	(0.938-3.682)	0.076
6th year	7.589	(3.469-16.601)	≤0.001	1.862	(0.891-3.891)	0.098	2.479	(1.178-5.218)	0.017
Encountered HBV patient									
Yes	1.667	(1.114-2.494)	0.013	2.249	(1.517-3.334)	≤0.001	1.262	(0.849-1.878)	0.250
No	Reference			Reference			Reference		
Extra courses about HBV									
Yes	NA			2.234	(1.174-4.250)	0.014	4.104	(1.983-8.494)	≤0.001
No				Reference			Reference		

*p*-value by multivariate logistic regression. Those variables labelled as “NA” were not statistically significant in the Chi-square. Hence, they were not included in this logistic regression. CI: confidence interval; OR: odds ratio; NA: not applicable.

## Data Availability

Data are available upon request to the corresponding author.

## Supplementary Materials

Supplementary Materials Supplementary file 1: Arabic and English versions of the questionnaire with correct answers.
